# linemodels: clustering effects based on linear relationships

**DOI:** 10.1093/bioinformatics/btad115

**Published:** 2023-03-02

**Authors:** Matti Pirinen

**Affiliations:** Institute for Molecular Medicine Finland (FIMM), Helsinki Institute of Life Science (HiLIFE), University of Helsinki, Helsinki 00014, Finland; Department of Mathematics and Statistics, University of Helsinki, Helsinki 00014, Finland; Department of Public Health, University of Helsinki, Helsinki 00014, Finland

## Abstract

**Summary:**

Estimation of effects of multiple explanatory variables on multiple outcome measures has become routine across life sciences with high-throughput molecular technologies. The *linemodels* R-package allows a probabilistic clustering of variables based on their observed effect sizes on two outcomes.

**Availability and implementation:**

An open source implementation in R available at github.com/mjpirinen/linemodels.

## 1 Introduction

In a recent study, COVID-19 Host Genetics Initiative (2022) asked whether each genetic variant associated with COVID-19 endpoints is actually associated with susceptibility to infection or severity of the disease. In another recent study, Hautakangas *et al.* (2022) assessed which migraine risk variants are specific to the subtype of migraine with aura. Both analyses were based on a Bayesian model comparison framework (Trochet *et al.*, 2019) which can handle correlated estimators due to, for example, overlapping samples between the analyses. The motivation for the *linemodels* package is to extend this framework to allow for modelling of arbitrary linear relationships between the variables and to provide an easy-to-use implementation in R.

In *linemodels*, the user specifies each model by three parameters:

- scale, i.e. the magnitude of the effects,
- slope, i.e. the multiplicative relationship between the expected values of the two effects, and
- correlation, i.e. the expected consistency with the expected values.

*linemodels* then estimates the membership probabilities of the variables in the given models, by taking into account the uncertainty in the effect estimates and the possible correlation of the two effect estimators. The package further allows for optimization of any set of model parameters using an expectation-maximization (EM) algorithm and estimation of the proportion parameters of the underlying mixture model using a Gibbs sampler.

## 2 Materials and methods

Let ${\hat{\beta }}_{ij}$ be the effect estimate of variable i=1,…,n on outcome *j *=* *1, 2, and ${\hat{\sigma }}_{ij}$ its estimated standard error. It is assumed that the effect estimators for different variables are independent while the two estimators of the same variable on the two outcomes may be correlated. Define, for k=1,…K, a line model $M_{k}$ via three parameters, $M_{k}=(s_{k},b_{k},r_{k})$, called scale *s_k_*, slope *b_k_*, and correlation *r_k_*. Intuitively, $M_{k}$ models the effects as centred around line $\beta_{i2}=b_{k}\beta_{i1}$, with larger of the two effects having prior standard deviation (‘scale’) of *s_k_* and the deviation from the line determined by the correlation coefficient *r_k_*. I first define the model for a diagonal case (slope = 1), and then use an orthogonal transformation to rotate the model to match the target slope.

For given $M_{k}=(s_{k},b_{k},r_{k})$, define the corresponding diagonal distribution of effect sizes as a bivariate Gaussian $N_{2}\left(0,D_{k}\right)$, where the covariance matrix is


$$D_{k}=\left(\begin{array}{cc} 1 & r_{k}\\ r_{k} & 1 \end{array}\right).$$

Let $T_{k}$ be the rotation matrix that transforms the diagonal line to the line with slope *b_k_*:


$$T_{k}=\left(\begin{array}{cc} \;\text{cos}(\alpha ) & -\text{sin}(\alpha )\\ \;\text{sin}(\alpha ) & \;\text{cos}(\alpha ) \end{array}\right),\;\text{where}\;\;\alpha =\arctan(b_{k})-\frac{\pi }{4}.$$

The prior distribution of the effect sizes according to model $M_{k}$ is then defined as $N_{2}\left(0,\Theta_{k}\right),$ where covariance matrix $\Theta_{k}=\frac{s_{k}^{2}}{m_{k}}T_{k}D_{k}T_{k}^{T}$ and normalization by $m_{k}=\max\{T_{k}D_{k}T_{k}^{T}\}$ confirms that the larger of the standard deviations of the two effects is *s_k_*. In *linemodels*, it is also possible to specify the prior distribution of the effects as a mixture of Gaussians, for example, to model heavier tails than in a Gaussian.

The observed effect size estimates are assumed a Gaussian distribution around the true effect sizes with covariance matrix


$${\hat{\Sigma }}_{i}=\left(\begin{array}{cc} {\hat{\sigma }}_{i1}^{2} & {\hat{\sigma }}_{i1}{\hat{\sigma }}_{i2}{\hat{\rho }}_{i}\\ {\hat{\sigma }}_{i1}{\hat{\sigma }}_{i2}{\hat{\rho }}_{i} & {\hat{\sigma }}_{i2}^{2} \end{array}\right),$$

where ${\hat{\sigma }}_{ij}$ is the standard error on outcome *j *=* *1, 2, and ${\hat{\rho }}_{i}$ describes how the two effect size estimators are correlated, for example, because of the sample overlap in the datasets from where the two effects have been estimated.

It follows that by combining the Gaussian prior with the Gaussian observation model, the marginal distribution for the observed effect size estimates ${\hat{\beta }}_{i}={\left({\hat{\beta }}_{i1},{\hat{\beta }}_{i2}\right)}^{T}$ under model $M_{k}$ is


$${\hat{\beta }}_{i}|M_{k}∼N_{2}\left(0,\Theta_{k}+{\hat{\Sigma }}_{i}\right).$$

When it is not feasible to fix every parameter of the line models before the analysis, *linemodels* provides an option to optimize any subset of the parameters using an EM algorithm.

**Membership probabilities.** Given *K* line models, ${\left(M_{k}\right)}_{k=1}^{K}$ and their prior probabilities $\pi ={\left(\pi_{k}\right)}_{k=1}^{K}$, one can estimate the posterior probabilities that each variable belongs to each of the models as


$$\text{Pr}(i∼M_{k}|{\text{Data}}_{i})=\frac{\pi_{k}N_{2}\left({\hat{\beta }}_{i}|0,\Theta_{k}+{\hat{\Sigma }}_{i}\right)}{\sum_{\ell =1}^{K}\pi_{\ell }N_{2}\left({\hat{\beta }}_{i}|0,\Theta_{\ell }+{\hat{\Sigma }}_{i}\right)}.$$

This calculation can be done separately for each variable and is implemented in *linemodels*.

If one does not want to pre-specify the numerical values of the prior probabilities of each model, one can set a prior distribution on π and estimate its posterior distribution together with the probabilistic assignment of variables into models. In *linemodels*, a prior distribution for this task is $\pi ∼\text{Dirichlet}(\delta_{1},\ldots ,\delta_{K})$, where the default values of the hyper-parameter are $\delta_{k}=\frac{1}{K}$ for each k=1,…,K. A Gibbs sampler to estimate the posterior distribution of this model is implemented in *linemodels*.

### 2.1 COVID-19 Host Genetics Initiative data

COVID-19 Host Genetics Initiative (2022) release 6 included genome-wide association studies (GWAS) of infection (INF, 114 516 SARS-Cov-2 infected versus 2 138 237 population controls) and hospitalization (HOS, 23 988 hospitalized for COVID-19 versus 2 834 885 population controls) that together identified 23 genome-wide significant lead variants (*P *<* *5e−8). The question is, for each lead variant, whether the variant is associated with susceptibility to infection or with severity of the disease.

**Figure 1:**
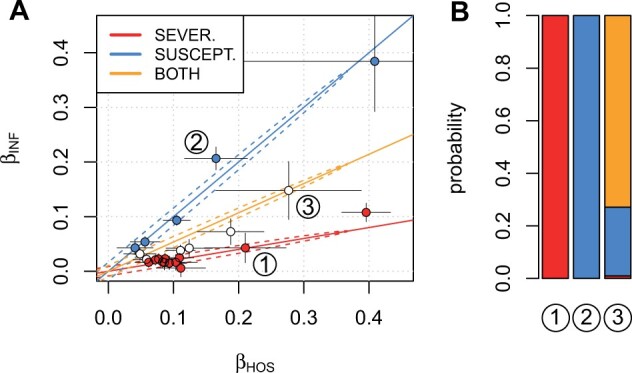
(A) COVID-19 HGI effect sizes from hospitalization (HOS) GWAS and infection (INF) GWAS for 23 variants with 95% confidence intervals. Three line models with 95% regions are shown by coloured lines. Variants with posterior probability >95% in one of the models are coloured according to the corresponding model. Three variants are labelled and posterior distributions of their assignment probabilities are shown in panel B

The two GWAS were nested (hospitalized patients were also cases in infection GWAS) and hospitalized patients were strongly enriched among infection GWAS cases compared to a random sample of the infected from the population. For these data, COVID-19 Host Genetics Initiative (2022) estimated that a pure susceptibility variant would show effect sizes $\beta_{\text{INF}}\approx \beta_{\text{HOS}}$ and a pure severity variant is expected to follow the relationship $\beta_{\text{INF}}\approx 0.2\cdot \beta_{\text{HOS}}$. Additionally, here I also consider a model for variants that may affect both susceptibility and severity, described by the line $\beta_{\text{INF}}\approx 0.535\cdot \beta_{\text{HOS}}$, where the slope is chosen to halve the angle between the lines of the other two models. Thus, I model these data with three line models (Fig. 1A):

- susceptibility effect (s=0.15,b=1,r=0.999),
- severity effect (s=0.15,b=0.2,r=0.999),
- both susceptibility and severity effects (s=0.15,b=0.535,r=0.999).

The chosen scale (*s *=* *0.15) assumes that most GWAS effects are small: about 95% of the effect sizes are below log odds ratio of 0.30. Correlation *r *=* *0.999 allows some deviation from the exact relationships (see dotted lines in Fig. 1) and thus adds robustness against model misspecification. The prior used for the proportion parameters of the three models was $\text{Dirichlet}\left(\frac{1}{3},\frac{1}{3},\frac{1}{3}\right)$.

## 3 Results

*linemodels* applied on the COVID-19 Host Genetics Initiative variants estimates that 64% (95% credible interval 42%, 82%) are pure disease severity variants, 25% (9%, 45%) are pure infection susceptibility variants, and the remaining 11% (0%, 31%) affect both. With posterior probability threshold of 0.95, 5 variants are affecting only susceptibility, 12 variants are affecting only severity, and 6 variants remain uncertain at this threshold (Fig. 1A). Figure 1B shows examples of a pure severity variant (1), a pure susceptibility variant (2), and a variant that potentially affects both phenotypes (3). See Supplementary Information for detailed results.

## 4 Conclusion

*linemodels* package provides tools for probabilistic clustering of variables based on linear relationships in their effect sizes on two outcomes.

## Supplementary Material

btad115_Supplementary_Data

## Data Availability

The data are available at www.covid19hg.org.
