# Supplementary material for: linemodels: clustering effects based on linear relationships
Source: Bioinformatics. 2023 Mar 2;39(3):btad115. doi: 10.1093/bioinformatics/btad115 (PMC10005595; doi:10.1093/bioinformatics/btad115)
Supplement: btad115_Supplementary_Data [file btad115_supplementary_data.pdf]

Supplementary Data of "linemodels: clustering effects based on linear relationships" by Matti Pirinen, University of Helsinki.

1. COVID-19 HGI analysis

Fig.1 of the main text shows a *linemodels* analysis of COVID-19 HGI data. The data are in Supplementary Table 1. The beta coefficients and their standard errors (SE) of the two GWAS (HOS and INF) originated from meta-analysis of GWAS results from logistic regression models across up to 60 participating cohorts. The recommended covariates to be used in the GWAS were age, age^2, sex, age × sex and the first 20 principal components of population genetic structure. Any other study-specific covariates to account for known technical artefacts may have been added by the cohorts. More details of the GWAS methods are given by COVID19-HGI (2021) in Nature 600: 472–477.

CHR	POS	RSID	REF	ALT	HOS_beta	HOS_se	HOS_pval	INF_beta	INF_se	INF_pval
1	155127096	rs148063273	C	T	0.2766	0.0569	1.19E-06	0.1479	0.0271	5.10E-08
1	155203736	rs67579710	G	A	-0.1035	0.0169	8.46E-10	-0.0163	0.0074	0.02872
3	45793925	rs73062389	G	A	0.1652	0.0247	2.17E-11	0.2064	0.0108	4.16E-82
3	45838989	rs35508621	T	C	0.3956	0.0191	4.55E-95	0.1077	0.0086	5.93E-36
3	101780431	rs17412601	T	C	-0.0563	0.0116	1.21E-06	-0.0537	0.0050	1.88E-26
6	31153455	rs111837807	T	C	0.1241	0.0182	9.09E-12	0.0423	0.0082	2.70E-07
6	33076153	rs2071351	A	G	0.0485	0.0127	0.00013	0.0318	0.0058	4.18E-08
6	41522644	rs41435745	G	C	0.1877	0.0261	6.45E-13	0.0724	0.0121	2.10E-09
9	133273813	rs505922	C	T	-0.1050	0.0107	9.36E-23	-0.0932	0.0048	4.31E-83
10	79946568	rs721917	A	G	0.0585	0.0105	2.73E-08	0.0217	0.0047	3.46E-06
11	1219991	rs35705950	G	T	-0.1112	0.0193	9.16E-09	-0.0057	0.0081	0.4853
11	34507219	rs766826	C	T	-0.0832	0.0119	2.99E-12	-0.0169	0.0051	0.00089
12	112936943	rs10774679	C	T	0.0772	0.0109	1.26E-12	0.0224	0.0049	4.27E-06
12	132564254	rs12809318	T	C	-0.0619	0.0108	1.07E-08	-0.0163	0.0048	0.00068
16	89196249	rs117169628	G	A	0.0860	0.0160	7.28E-08	0.0159	0.0069	0.02164
17	45707983	rs61667602	T	C	-0.0935	0.0136	5.28E-12	-0.0147	0.0060	0.01415
17	49863303	rs77534576	C	T	0.2103	0.0319	4.67E-11	0.0427	0.0139	0.00205
19	4719431	rs2109069	G	A	0.1106	0.0116	2.01E-21	0.0380	0.0052	2.49E-13
19	10355447	rs11085727	C	T	0.0870	0.0117	1.23E-13	0.0229	0.0052	1.14E-05
19	48867352	rs4801778	G	T	-0.0410	0.0142	0.00391	-0.0428	0.0062	7.39E-12
19	50379362	rs1405655	T	C	0.0721	0.0110	4.87E-11	0.0207	0.0049	2.21E-05
21	33242905	rs13050728	T	C	-0.1091	0.0111	1.25E-22	-0.0247	0.0050	6.74E-07
23	15602217	rs190509934	T	C	-0.4087	0.1176	0.00051	-0.3842	0.0469	2.51E-16

Supplementary Table 1. Input data of the COVID-19 HGI analysis of the main text. Columns: CHR = chromosome, POS = position in GRCh38, RSID = reference SNP id, REF = reference allele, ALT = alternative allele, HOS = hospitalization GWAS, INF = infection GWAS, beta = logistic regression effect size (log odds-ratio) for ALT allele, se = standard error of beta, pval = P-value of beta.

The model probabilities from the *linemodels* analysis are in Supplementary Table 2.

CHR	POS	RSID	Pr_SEV	Pr_SUC	Pr_MID
1	155127096	rs148063273	0.0099	0.2611	0.729
1	155203736	rs67579710	1	0	0
3	45793925	rs73062389	0	1	0
3	45838989	rs35508621	1	0	0
3	101780431	rs17412601	0	0.9951	0.0049
6	31153455	rs111837807	0.9052	0	0.0948
6	33076153	rs2071351	0.0112	0.5168	0.472
6	41522644	rs41435745	0.3772	3.00E-04	0.6225
9	133273813	rs505922	0	1	0
10	79946568	rs721917	0.785	0.0053	0.2097
11	1219991	rs35705950	0.9999	0	1.00E-04
11	34507219	rs766826	0.9998	0	2.00E-04
12	112936943	rs10774679	0.9903	0	0.0097
12	132564254	rs12809318	0.9888	1.00E-04	0.0111
16	89196249	rs117169628	0.9984	0	0.0016
17	45707983	rs61667602	1	0	0
17	49863303	rs77534576	1	0	0
19	4719431	rs2109069	0.8974	0	0.1026
19	10355447	rs11085727	0.9987	0	0.0013
19	48867352	rs4801778	0	0.9588	0.0412
19	50379362	rs1405655	0.9862	0	0.0138
21	33242905	rs13050728	1	0	0
23	15602217	rs190509934	0	0.9997	3.00E-04

Supplementary Table 2. Results of the COVID-19 HGI analysis of the main text. Columns: CHR = chromosome, POS = position in GRCh38, RSID = reference SNP id, Pr = Posterior probability of a model, SEV = model for a pure severity effect, SUC = model for a pure infection susceptibility effect, MID = model for both a severity and a susceptibility effect.

The R code to reproduce the analysis is available at https://raw.githubusercontent.com/mjpirinen/linemodels/main/linemodels_examples.R

2. Availability of linemodels package and instructions

The *linemodels* package has been written in R. The source codes, a detailed manual describing the functionalities and mathematical and technical details of the method are available at https://github.com/mjpirinen/linemodels
